# Intensifying the Hormonal and Radiation Treatment in Prostate Cancer Presenting With Bulky Pelvic Lymph Nodes: An Opportunity for a New Paradigm

**DOI:** 10.7759/cureus.51518

**Published:** 2024-01-02

**Authors:** Agha H Khan, Nadeem Pervez, Muhammad S Usmani, Houda S Almusalhi, Munjid Al Harthy, Layth Mula-Hussain

**Affiliations:** 1 Radiation Oncology, McGill University, Montreal, CAN; 2 Radiation Oncology, Sultan Qaboos Comprehensive Cancer Care and Research Centre, Muscat, OMN; 3 Nuclear Medicine, Sultan Qaboos Comprehensive Cancer Care and Research Centre, Muscat, OMN; 4 Radiation Oncology, Sultan Qaboos University, Muscat, OMN; 5 Medical Oncology, Sultan Qaboos Comprehensive Cancer Care and Research Centre, Muscat, OMN; 6 Department of Radiation Oncology, Faculty of Medicine, Dalhousie University, Halifax, CAN

**Keywords:** intensity-modulated radiation therapy (imrt), radical treatment, hypofractionated rt, psma scan, radiotherapy, duo hormonal therapy, locally advanced, bulky lymph nodes, prostate cancer

## Abstract

Locally advanced prostate cancer may rarely present with bulky pelvic lymph nodes without distant metastasis. Patients may be treated with curative intent. Dual hormonal therapy including luteinizing hormone-releasing hormone agonist in combination with abiraterone or enzalutamide can be utilized neoadjuvantly to shrink bulky disease. This can be followed by radical doses of radiotherapy. This intensified treatment is tolerable. Prostate-specific membrane antigen scan can be utilized to assess staging and treatment response. Here, we present a case of a non-metastatic locally advanced prostate cancer with bulky pelvic lymph nodes. The patient was treated neoadjuvantly with dual hormonal therapy followed by radical doses of radiotherapy. The patient tolerated the treatment well and had a promising early response.

## Introduction

Prostate cancer is one of the most prevalent cancers in the male population and one of the leading causes of mortality in some regions of the world, according to GLOBOCAN data [1]. Pelvic nodal involvement at presentation in prostate cancer is uncommon. It is an important factor in determining the stage and prognosis of the disease [2]. Patients with involved lymph nodes at presentation are at an increased risk of recurrence and progression. Optimal treatment strategies for this population are still evolving with multiple options including systemic and local treatment. In our case study, we intend to report the treatment strategy of a prostate cancer patient with bulky lymph nodes in the pelvis at presentation. The patient was initially treated by dual hormone treatment followed by locoregional curative doses of radiation after a complete metabolic response shown on functional imaging.

## Case presentation

A 74-year male, with no comorbidities, presented to the emergency department with an incidental fall with backache. Subsequent imaging investigation with computed tomography (CT) scan with contrast found a trauma-related collapse fracture of the D4 vertebra (non-metastatic). It also showed enhancing lesions in the prostate gland measuring 4.2 x 3.2 x 3.0 cm and multiple enlarged regional nodes including pre-sacral, external, internal, and common iliac lymph, with the largest on the right measuring 8 x 7 x 4 cm. A magnetic resonance imaging (MRI) of the pelvis showed a suspicious nodule in the peripheral zone of the prostate gland with multiple enlarged pelvic and mesorectal lymph nodes, with the largest on the right side measuring 6.9 x 4.2 cm and on the left side measuring 5.4 x 4 cm. Prostate-specific antigen (PSA) level was more than 100 ng/mL. Transrectal ultrasound (TRUS) biopsy of the prostate confirmed invasive acinar adenocarcinoma with Gleason grade group 5 (4+5 = 9) in all cores.

**Figure 1:**
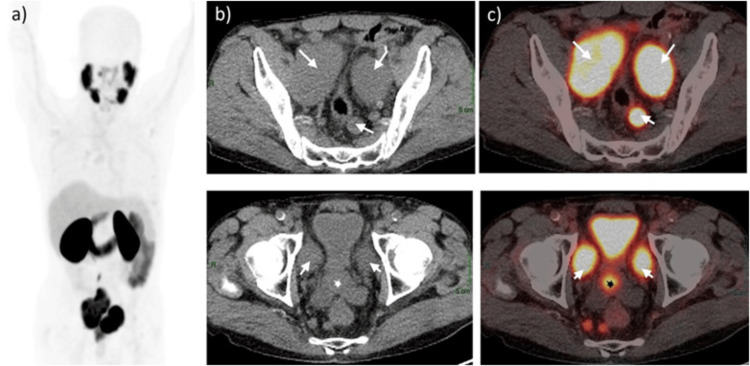
Baseline gallium-68-prostate-specific membrane antigen positron emission tomography/computed tomography (68Ga-PSMA-PET/CT). (a) Maximum intensity projection image showing prostate gland and multiple pelvic lymph nodes tracer uptake. (b) Non-contrast CT showing enlarged prostate gland and multiple enlarged pelvic lymph nodes. (c) Fused PET/CT images show large tracer-avid lesion on the right lobe of the prostate gland extending up to the seminal vesicle (marked with *) with standardized uptake value (SUV) maximum of 12.4. There are multiple enlarged lymph nodes (marked with arrows) in the perirectal and bilateral external iliac regions (SUV max of 28.8). The largest lymph node with mass formation is seen in the right external iliac region, extending superiorly up to the right common iliac region measuring approximately 7.6 (anteroposterior) × 4.6 (transverse) cm in size.

The patient was discussed during a site-specific multi-disciplinary team (MDT) meeting. A prostate-specific membrane antigen positron emission tomography (PSMA-PET) with Gallium (Ga)-68 was performed for staging as per MDT suggestion. The scan showed PSMA-avid lesions in the prostate gland, extending up to the seminal vesicles and multiple enlarged tracer-avid pelvic lymph nodes. The largest lymph node was in the right external iliac region, extending superiorly up to the right common iliac region measuring approximately 7.6 cm anteroposteriorly × 4.6 cm transverse dimensions with standardized uptake value (SUV) maximum of 28.8 (Figure 1).

**Figure 2:**
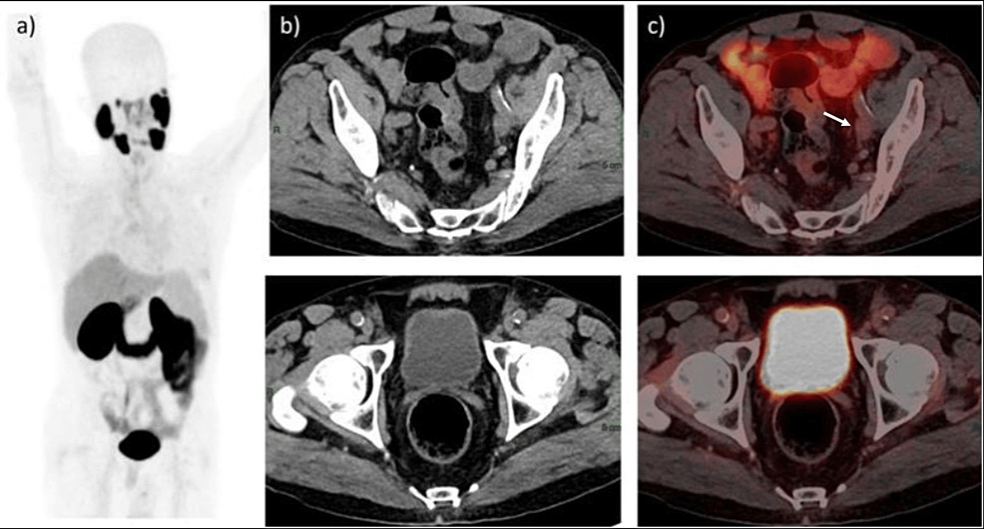
Post-therapy gallium-68-prostate-specific membrane antigen positron emission tomography/computed tomography (68Ga-PSMA-PET/CT). (a) Maximum intensity projection image. (b) Non-contrast CT. (c) Fused PET/CT images show complete resolution of prior PSMA avid seen in the prostate gland extending to the seminal vesicle. No abnormal uptake is seen in the prostate. Near-complete resolution of prior 68Ga-PSMA-avid perirectal and bilateral external iliac lymph nodes. Small bilateral external iliac lymph nodes are seen with no significant tracer uptake, with the largest measuring 1.3 x 1.0 cm at the left external iliac region (white arrow).

**Figure 3:**
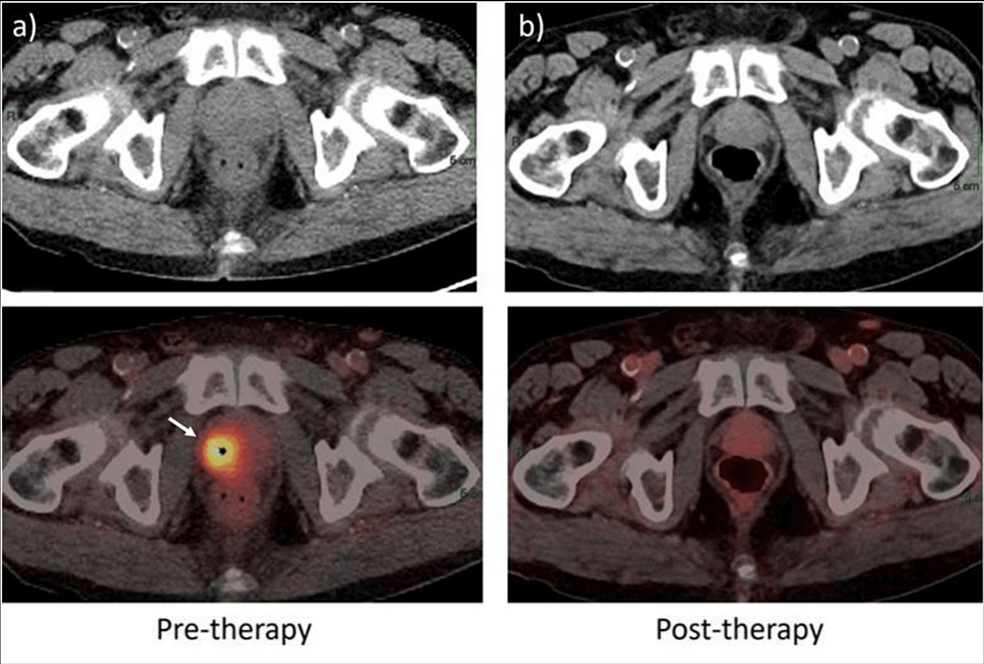
Baseline gallium-68-prostate-specific membrane antigen positron emission tomography/computed tomography (68Ga-PSMA-PET/CT) shows a large tracer-avid lesion in the prostate gland’s right lobe extending up to the seminal vesicles in the midline with standardized uptake value maximum of 12.4 (white arrow and star). Post-therapy 68Ga-PSMA-PET/CT show complete resolution of prior PSMA-avid seen in the prostate gland.

**Figure 4:**
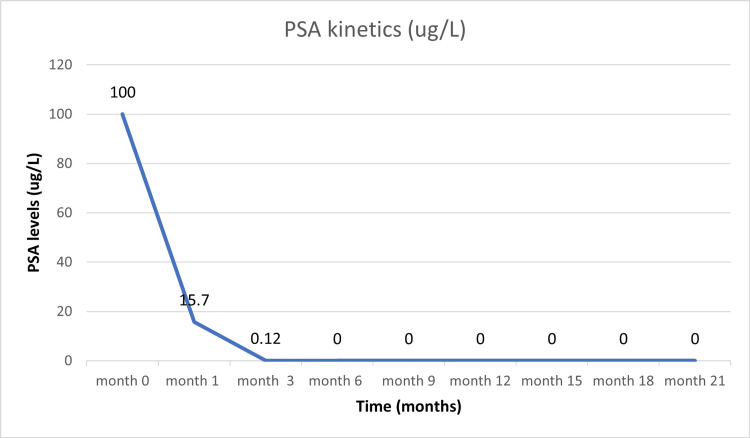
Prostate-specific antigen (PSA) kinetics displays a rapid fall in serum PSA levels with the start of systemic treatment and continued to be undetectable at the 21-month follow-up since the beginning of treatment (systemic).

The case was again discussed in MDT and the patient was offered and started on goserelin 10.8 mg injected subcutaneously every three months, abiraterone acetate 1,000 mg, and prednisone 5 mg daily orally. CT of the chest, abdomen, and pelvis was performed after the second dose of goserelin (at six months) before radiation treatment and showed interval reduction in the size of pelvic nodes (to the size of 4.1 × 2.4 cm was 9.7 x 4.6 cm), followed by PSMA-PET which showed complete resolution of prior Ga-68 PSMA-avid lesion in the prostate and pelvic lymph nodes. PSA levels dropped to undetectable levels of <0.01 ng/L at six months after starting hormonal therapy (Figures 2-4).

**Figure 5:**
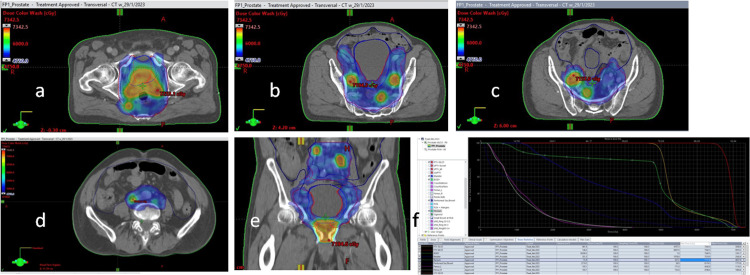
Radiotherapy treatment planning CT images showing targets and organs at risk (color lines) and radiation dose clouds (increasing dose from blue to red). (a) Axial image at the prostate gland level. (b and c) Axial images at the mid-pelvis level. (d) Axial image at the upper pelvis level. (e) Coronal plane. (f) Dose-volume histograms.

The patient was again discussed in MDT and it was decided to offer definitive radiation treatment due to the hormonal therapy response. He was offered a previously reported dose of 50 Gray (Gy) in 25 fractions to elective pelvic lymph nodes (common iliac chain, external (upper) and internal Iliac chains, pre-sacral, mesorectal, and obturator chain of lymph nodes) and simultaneous integrated boost (SIB) doses to the prostate, seminal vesicles, and residual gross nodal disease to a dose of 68 Gy in 25 fractions (moderately hypofractionated doses) over five weeks. Treatment planning was performed on Eclipse™(Varian Oncology Systems Inc. CA, USA), treatment was delivered using volumetric-modulated arc therapy, and treatment set-up was verified using cone beam CT before each treatment (Figure 5).

The patient tolerated the treatment well and had no gastrointestinal (GI) or genitourinary (GU) toxicities during treatment. At the three-month follow-up, there were no complaints except mild urinary frequency (no medication prescribed). The patient was offered and agreed to continue goserelin for three years and abiraterone acetate with prednisone for two years. The patient was followed up every three months. He completed 21 months of follow-up after starting hormonal therapy and eight months after completion of radiotherapy with no late GI and GU toxicities. His current serum PSA levels remain undetectable at <0.01 ng/L (Figure 4).

## Discussion

Historically, node-positive prostate cancer patients were considered palliative and usually received only hormonal therapy. More recently, these patients are offered a combination of dual hormonal therapy with radical doses of radiotherapy for the gross disease as conformal volumes, intensity-modulated radiation therapy (IMRT), and image-guided radiation therapy (IGRT) improve treatment tolerance.

Radiation Therapy Oncology Group trial 8531 showed 10 years of overall (OS) survival benefit by 10% (from 39 to 49 %) and disease-specific survival by 6% (from 78% to 84%) in 1997 with the addition of pelvic radiation to hormone treatment [3]. Similar results were published in the subset analysis by Pilepich et al. in 2005 who reported 10% and 4% improvement in progression-free survival (PFS) on five and nine years of follow-up, respectively, when radiation was added with hormone therapy in node-positive patients [4]. The stampede group showed improvement in OS from 53% to 81% at nine years when radiation was added to the systemic treatment of node-positive patients [5]. However, these studies offered radical doses to the prostate gland and not to the grossly involved pelvic lymph node (PLN). A phase II prospective study in high-risk prostate cancer where some patients presented with involved PLN (non-bulky) was reported by Cross Cancer Institute, Canada. These patients were treated with moderately hypofractionated doses of 68 Gy to gross disease including involved nodes and 45-50 Gy to elective volumes in 25 fractions using the SIB technique in a single-phase plan. This study was different in terms of delivering radical doses to the involved gross PLN with promising results [6]. In contrast, all above-mentioned studies did not deliver radical doses to the grossly involved PLN.

The introduction of functional imaging with specific tracers has rapidly progressed resulting in the adaptation of personalized or tailored approaches for managing a cancer patient. It has not only increased the diagnostic specificity but also helps in detecting early responses and predicting prognosis [7]. One of many tracers in prostate cancer is Ga-68 PSMA-PET. Pietro et al. reported 30% (76.9% vs. 46%) better accuracy in detecting nodal metastasis when compared with conventional CT imaging with technetium 99m bone scan [8]. Our patient had PSMA-PET/CT imaging for staging workup and for response assessment which has not been reported in the published literature for bulky prostate cancer yet.

Systemic options for metastatic prostate cancer are accumulating with time. Multiple trials have shown the addition of chemotherapy, and the new generation of hormones have improved patient-related outcomes. The CHAARTED trial showed benefit by 10 months (from 47.2 to 57.6 months) in patients where docetaxel was added with hormone treatment [9]. Davis et al. reported an 8% (from 72% to 80%) OS advantage at the three-year follow-up; moreover, PSA PFS was double in the enzalutamide group [10]. The LATITUDE trial also reported OS and radiographic PFS benefits with the addition of abiraterone acetate and prednisone to hormone treatment, with significant improvement in OS of up to 19 months (from 36.5 to 53.3 months) [11]. With this emerging data, these newer agents are now the standard of care for all prostate cancer patients representing this cohort. Our patient was offered abiraterone with prednisone and goserline because of bulky nodal disease with near-complete metabolic responses on the PSMA-PET/CT scan.

To our knowledge, only two case reports have been published so far reporting outcomes of bulky nodal disease for prostate cancer. None of these case reports has utilized the PSMA-PET/CT scan and dual hormonal therapy as utilized in our case report [12,13].

Based on our information, this is the first case report from our region reporting prostate cancer patients with bulky PLN, staging, and treatment response assessed by PSMA-PET scan, treated by dual hormonal therapy and moderately hypofractionated radical radiotherapy doses.

Limitations of our report are single case, retrospective analysis, and short follow-up. The patient remains on continued follow-up. We are planning to report long-term follow-up findings in the future.

## Conclusions

Prostate cancer can rarely present with bulky pelvic lymph nodes. Site-specific MDT management provided an opportunity to adopt the personalized treatment approach to this patient. PSMA-PET imaging played an important role in staging and treatment response assessment. Dual hormonal therapy with goserelin and abiraterone acetate with prednisone resulted in complete metabolic and radiological responses on imaging. Moderately hypofractionated radical radiation doses were safely delivered using IMRT and IGRT techniques. Systemic and radiotherapy treatments were tolerated well. Early results are encouraging. The patient is under surveillance for longer-term outcomes.
